# Composition Design of a Novel High-Temperature Titanium Alloy Based on Data Augmentation Machine Learning

**DOI:** 10.3390/ma18133099

**Published:** 2025-06-30

**Authors:** Xinpeng Fu, Boya Li, Binguo Fu, Tianshun Dong, Jingkun Li

**Affiliations:** 1 State Key Laboratory of High Performance Roll Materials and Composite Forming, School of Materials Science and Engineering, Hebei University of Technology, Tianjin 300401, China; fuxp_1995@163.com (X.F.); dongtianshun111@163.com (T.D.); tjjk_zy@126.com (J.L.); 2 Hebei Key Laboratory of New Functional Materials, School of Materials Science and Engineering, Hebei University of Technology, Tianjin 300401, China; 3 School of Materials and Chemical Technology, Institute of Science Tokyo, Tokyo 152-8552, Japan

**Keywords:** high-temperature titanium alloy, machine learning, data augmentation, microstructure, mechanical property

## Abstract

The application fields of high-temperature titanium alloys are mainly concentrated in the aerospace, defense and military industries, such as the high-temperature parts of rocket and aircraft engines, missile cases, tail rudders, etc., which can greatly reduce the weight of aircraft while resisting high temperatures. However, traditional high-temperature titanium alloys containing multiple types of elements (more than six) have a complex impact on the solidification, deformation, and phase transformation processes of the alloys, which greatly increases the difficulty of casting and deformation manufacturing of aerospace and military components. Therefore, developing low-component high-temperature titanium alloys suitable for hot processing and forming is urgent. This study used data augmentation (Gaussian noise) to expedite the development of a novel quinary high-temperature titanium alloy. Utilizing data augmentation, the generalization abilities of four machine learning models (XGBoost, RF, AdaBoost, Lasso) were effectively improved, with the XGBoost model demonstrating superior prediction accuracy (with an R^2^ value of 0.94, an RMSE of 53.31, and an MAE of 42.93 in the test set). Based on this model, a novel Ti-7.2Al-1.8Mo-2.0Nb-0.4Si (wt.%) alloy was designed and experimentally validated. The UTS of the alloy at 600 °C was 629 MPa, closely aligning with the value (649 MPa) predicted by the model, with an error of 3.2%. Compared to as-cast Ti1100 and Ti6242S alloy (both containing six elements), the novel quinary alloy has considerable high-temperature (600 °C) mechanical properties and fewer components. The microstructure analysis revealed that the designed alloy was an α+β type alloy, featuring a typical Widmanstätten structure. The fracture form of the alloy was a mixture of brittle and ductile fracture at both room and high temperatures.

## 1. Introduction

Titanium alloys are widely used in fields such as aerospace, medicine, defense and military industry due to their low density, high specific strength, and excellent corrosion resistance. The rapid development of the aerospace industry has spotlighted the requirement for advanced high-temperature titanium alloys [1,2,3]. Currently, the more typical high-temperature titanium alloys at 600 °C are IMI834 and Ti1100, both of which belong to the Ti-Al-Sn-Zr-Mo-Si alloy system [4,5,6]. With the development of aerospace technology, titanium alloys need to withstand higher temperatures (more than 600 °C) on the basis of structural weight reduction. At the same time, titanium alloy components are increasingly showing trends of large size, thin-walled surfaces, variable thickness, and overall structural development, which poses new challenges for the processing and manufacturing of titanium alloys. However, there is a lack of systematic research on the composition design of high-temperature titanium alloys that combine heat resistance and formability both domestically and internationally. The development of traditional high-temperature titanium alloys is achieved by the addition of rare earth elements (Y, Er, Nd) or β-stabilizing elements (Nb, Ta, W) on the basis of the above-mentioned system, and performance is improved through alloying [7,8,9,10]. However, the addition of too many components has a complex impact on the solidification, deformation, and phase transformation processes of the alloys, which greatly increases the difficulty of material casting and deformation manufacturing. This approach also faces diminishing returns in performance improvement, increasing material costs, and recycling complexities [11,12]. Against this background, this study aimed to break the traditional system of high-temperature titanium alloys (Ti-Al-Sn-Zr-Mo-Si) and develop a novel high-temperature titanium alloy that contained only five elements. By optimizing the types and contents of alloying elements, the costs and dependence on scarce resources can be reduced without affecting performance.

The selection of elements underwent rigorous analysis and screening. The Al element is the most commonly used element in high-temperature titanium alloys. The addition of the Al element can enhance the thermal stability of titanium alloys and reduce the alloy density. Moreover, due to its strong strengthening effect and low cost, the Al element is usually used for manufacturing low-cost titanium alloys [13,14]. The Mo element can effectively enhance the strength of the alloy at room and high temperatures by strengthening the β-phase [15,16]. The Nb element is a relatively weak β-stabilizing element, which has an electronegativity similar to that of Ti and exhibits a high solid solubility in the α-phase. As such, it can effectively exert solid solution strengthening effects on both the α-phase and the β-phase simultaneously [17,18,19]. The Si element is also a commonly added element in high-temperature titanium alloys, which contributes to solid solution strengthening and forms silicides that pin dislocations and boundaries, further elevating alloy strength [20,21,22]. Meanwhile, the formation of Ti_5_Si_3_ silicide with a D8_8_ hexagonal structure (lattice parameters a = 0.7444 nm and c = 0.5143 nm) features a high melting point (2130 °C) and low density (4.32 g/cm^3^). Its thermal expansion coefficient is similar to that of titanium, thereby improving the creep resistance of the alloy [23,24]. Therefore, Al, Mo, Nb and Si were selected as the constituent elements of the alloy in this study.

The empirical trial-and-error method has the disadvantages of high cost and long material design cycle. For instance, exploring just 10 content levels across the four elements Al, Mo, Nb, and Si would generate 10,000 potential combinations, which severely restricts the development of novel alloys. With the rapid development of artificial intelligence (AI) technology and the accumulation of experimental data, the machine learning (ML) method has shown great potential in material research and development [25,26,27]. Liu et al. established four ML models (Adaptive Boosting, Light Gradient Boosting Machine, Voting, and Stacking) for the design of metastable β-titanium alloys, among which the Light Gradient Boosting Machine model had the optimal prediction accuracy. Based on this model, a metastable β-titanium alloy (Ti-5.5Cr-5Al-4Mo-3Nb-2Zr) was designed, and the experimental values of ultimate tensile strength (UTS), yield strength (YS), and elongation (EL) were in good agreement with the values predicted by the model [28]. Wu et al. used an Artificial Neural Network (ANN) model to design a Ti-12Nb-12Zr-12Sn alloy. This alloy met the requirements of biocompatibility, low modulus, and low cost, and was expected to be used in orthopedics and prosthetic implantation [29]. Oh et al. constructed a dataset of Mn content, solution treatment temperature, UTS, and EL to train an ANN model. Based on this model, a low-cost TRIP titanium alloy, Ti-4Al-2Fe-1.4Mn, was developed. After solution treatment at 883 °C, this alloy had an ultra-high specific strength (289 MPa·cm^3^/g) and excellent elongation (34%) [30]. However, the potential of ML in high-temperature titanium alloy design remains untapped.

The purpose of this study is to find a design method that combines low-component and high-heat-resistant temperature titanium alloys. This study established a dataset of the alloy composition, test temperature, and UTS of high-temperature titanium alloys, and data augmentation was carried out on the original dataset. The predictive performance of four ML models was systematically evaluated, and an optimal ML model was used to develop a novel high-temperature titanium alloy. Beyond high predictive accuracy, the model’s efficacy was validated through alloy preparation and performance testing, providing a new idea for the development of novel high-temperature titanium alloys. The novel titanium alloy successfully reduces the number of alloying elements while ensuring high performance. And this is of great significance for promoting the application of high-temperature titanium alloy castings in the aerospace industry.

## 2. Material Design and Experiment

### 2.1. Alloy Design Framework

**Figure 1:**
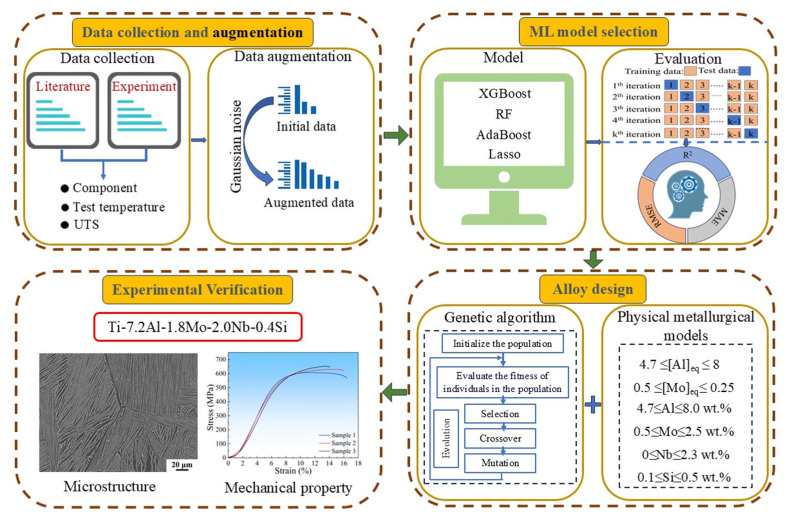
ML design scheme for novel high-temperature titanium alloy.

Figure 1 schematically illustrates the design process of a novel high-temperature titanium alloy through ML methods. As shown in Figure 1, the first step is data collection and augmentation: through a literature review, an original dataset encompassing alloy compositions, testing temperatures, and UTS was constructed, and an augmented dataset was obtained through data augmentation. The second step is ML model selection: the generalization ability and prediction accuracy of four ML models were compared to determine the optimal ML model. The third step is alloy design: the physical metallurgy model was combined with the genetic algorithm for composition design. The fourth step is experimental verification: the properties and microstructure of the alloy were analyzed to verify the effectiveness of the design scheme in this study.

### 2.2. Dataset Establishment

In this study, a dataset containing 114 samples was established through consulting the literature, as shown in Table 1. The dataset consists of three parts, namely, the alloy composition and testing temperature as input features, and the UTS as the output feature. The alloy composition includes ten elements, Al, Sn, Zr, Mo, Si, Nb, Ta, W, Y, and V. The testing temperature ranges from 20 °C to 750 °C, and the UTS ranges from 381 MPa to 1328 MPa. In order to ensure the scientific rigor and applicability of the data, the following data screening criteria were set: (1) The alloy system was limited to near-α type or α+β type titanium alloys with high-temperature application potential. (2) All alloys involved in the data were prepared by casting forming. (3) The collected values of the UTS were all the properties of the alloys in the as-cast condition, excluding the interference of subsequent thermomechanical treatments (plastic deformation, heat treatment).

### 2.3. Data Augmentation

To further enhance the data quality, this study attempted to achieve data augmentation by adding noise points to the original data [31]. This approach is particularly relevant to material science because it simulates the uncontrollable errors in practical alloy design. For example, during the smelting of the alloy, the loss of raw materials can lead to a deviation between the actual composition and the nominal composition. In the application of material design, Ye et al. carried out data augmentation through the Gaussian noise method, achieving accurate prediction of the phases and hardness of high-entropy alloys, which demonstrated its effectiveness [32]. The enhancement of data with Gaussian noise can be achieved through Equation (1) [33]:

(1) $$x_{aug}^{i}=x+\sigma \cdot \varepsilon^{\left(i\right)},i=1,2,\ldots \ldots ,\;n$$

where $x_{aug}^{i}$ is the *i*th augmented data point, x is the original data, σ is the standard deviation of the Gaussian noise, $\varepsilon^{\left(i\right)}$~N (0, 1) indicates that the randomly sampled variable follows a normal distribution, and *n* is the number of augmented data points generated for each original data point. In this study, σ and *n* were set to 0.01 and 1, respectively.

### 2.4. Model Establishment and Evaluation

**Figure 2:**
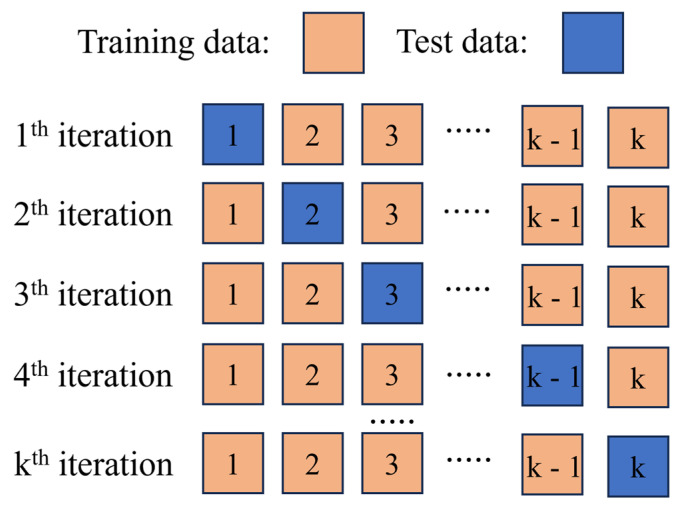
Schematic diagram of 10-fold cross-validation.

According to the “No Free Lunch Theorem” proposed by Wolpert, there is no single algorithm that can consistently outperform all other algorithms across all possible problems or datasets [34]. Therefore, in this study, four different ML models, namely, eXtreme Gradient Boosting (XGBoost), Random Forest (RF), Adaptive Boosting (AdaBoost), and Least absolute shrinkage and selection operator (Lasso), were established. Firstly, the impact of data augmentation on the performance of the ML model was evaluated through 10-fold cross-validation. Figure 2 is a schematic diagram of 10-fold cross-validation. Its principle is as follows: the dataset is divided into 10 non-overlapping subsets. In each iteration, 9 of these subsets are selected as the training set to train the model, and the remaining 1 subset is used as the test set to evaluate the performance of the model. This process is repeated 10 times to ensure that each subset is used as the test set. Finally, the average value of the coefficient of determination (R^2^) of the 10 validations is calculated to obtain the 10-fold cross-validation score. The closer the score is to 1, the better the generalization ability of the model.

Next, the four ML models were trained and tested to determine the optimal model. The ratio of the test set to the training set was 1:4. The prediction accuracy of the four ML models was evaluated using the root mean squared error (RMSE), the mean absolute error (MAE), and the coefficient of determination (R^2^). The equation is as follows:

(2) $$RMSE=\sqrt{\frac{1}{n}\sum_{i=1}^{n}{\left(y_{i}-f\left(x_{i}\right)\right)}^{2}}$$

(3) $$MAE=\frac{1}{n}\sum_{i=1}^{n}\left|\left(y_{i}-f\left(x_{i}\right)\right)\right|$$

(4) $$R^{2}=1-\frac{\sum_{i=1}^{n}{\left(y_{i}-f(x_{i})\right)}^{2}}{\sum_{i=1}^{n}{\left(y_{i}-\bar{y}\right)}^{2}}$$

where *n* is the number of samples in the dataset, $\bar{y}$ is the average value of all experimental values in the dataset, and *y_i_* and *f*(*x_i_*) are the experimental value of each sample in the dataset and the corresponding predicted value of the ML model, respectively.

### 2.5. Alloy Design

The content ranges of four elements, Al, Mo, Nb and Si, were determined by combining physical metallurgy models such as Aluminum equivalent ([Al]_eq_) and Molybdenum equivalent ([Mo]_eq_) with domain knowledge. By imposing compositional constraints, the design efficiency of the ML model can be improved, and the reliability of the design results can be ensured [29,35]. The genetic algorithm was employed for composition screening, and the optimal alloy composition along with its corresponding predicted value of the UTS was obtained. The parameter settings of the genetic algorithm were as follows: the population size was set to 50, the number of generations was set to 1000, the crossover probability was set to 0.8, and the mutation probability was set to 0.2.

### 2.6. Experiment

**Figure 3:**
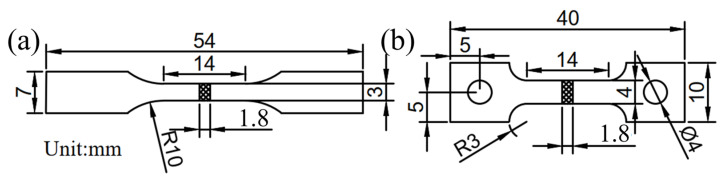
Dimensions of the alloy tensile specimens: (**a**) room temperature; (**b**) 600 °C.

The alloy was prepared by using a vacuum suspension melting furnace equipped with a water-cooled copper crucible, and the ultimate vacuum degree of the melting furnace was 10^−2^ Pa. Pure metals of Ti (99.90 wt.%), Al (99.99 wt.%), Mo (99.95 wt.%), Nb (99.95 wt.%), and Si (99.95 wt.%) were used as raw materials. To ensure the uniformity of the composition, the alloy ingot was melted three times. Alloy phases were examined by an X-ray diffractometer (XRD) with Cu Kα X-radiation (Rigaku Smartlab 9 kW, Akishima, Japan). The XRD sample was scanned over a range of 20°~80° with a scanning speed of 10°/min. The microstructure and tensile fracture surface of the alloy were observed using a scanning electron microscope (SEM, Hitachi SU3800, Hitachinaka, Japan). The microstructure sample was chemically etched using Kroll reagent (HF:HNO_3_:H_2_O = 1:2:7, vol.%). The room-temperature tensile property of as-cast alloy was tested using an Instron-5982 (Boston, MA, USA) universal electronic testing machine with a tensile rate of 1 mm/min. The tensile property of as-cast alloy at 600 °C was tested using a CMT-5205 (Ningbo, China) electronic universal testing machine with a tensile rate of 0.6 mm/min and a holding time of 10 min according to the GB/T 228.2-2015 standard [36]. According to the ASTM: E8/E8M-16 standard [37], a 15 mm gauge length extensometer was used to measure the strain to exclude the influence of the elastic strain of the grips. The yield strengths were calculated using the 0.2% strain offset method, as described in [38]. To ensure the reliability and repeatability of the data, three specimens were tested under each condition. The dimensions of the tensile specimens are shown in Figure 3.

## 3. Results and Discussion

### 3.1. Gaussian Enhancement

**Figure 4:**
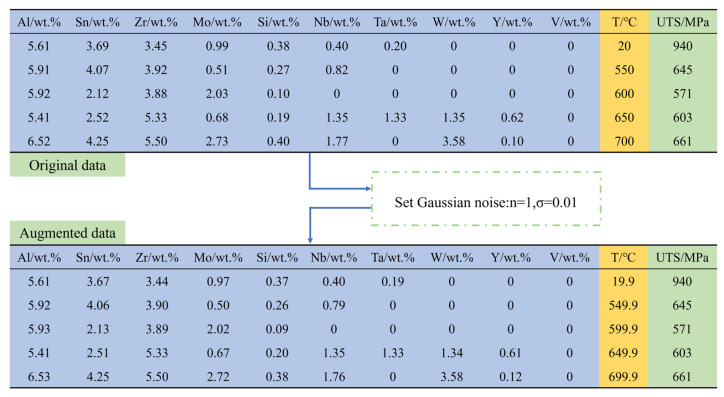
Schematic diagram of adding Gaussian noise to original data.

**Figure 5:**
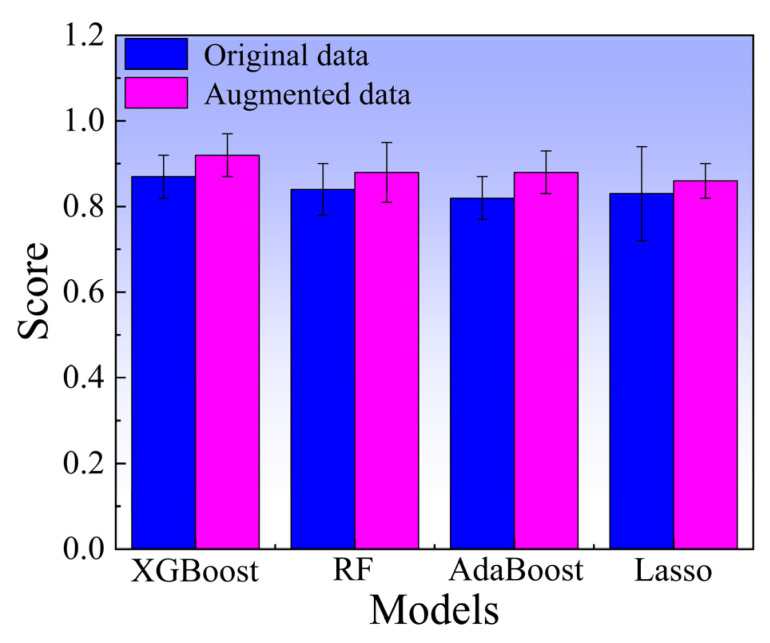
Comparison between the original dataset and the augmented dataset.

Figure 4 schematically illustrates the process of introducing Gaussian noise into the original dataset. The data in three different colors represent the alloy composition, testing temperature, and UTS, respectively. The data in the first five columns show that by introducing Gaussian noise into the original data values, the so-called “actual values” or noisy samples could be generated (the complete original data and augmented data can be found in Supplementary Tables S1 and S2). This method effectively doubles the size of the dataset. In order to evaluate the impact of data augmentation on the performance of the ML models, the performance of the four ML models was evaluated using 10-fold cross-validation. As shown in Figure 5, after data augmentation, the 10-fold cross-validation scores of the XGBoost, RF, AdaBoost, and Lasso models increased from 0.87, 0.84, 0.82, and 0.83 to 0.92, 0.88, 0.88, and 0.86, respectively. This enhancement can be attributed to the increased data diversity by data augmentation. The expanded dataset enabled the models to learn more features during the training process, thereby enhancing the generalization ability of the model.

### 3.2. Selection of Optimal ML Model

**Figure 6:**
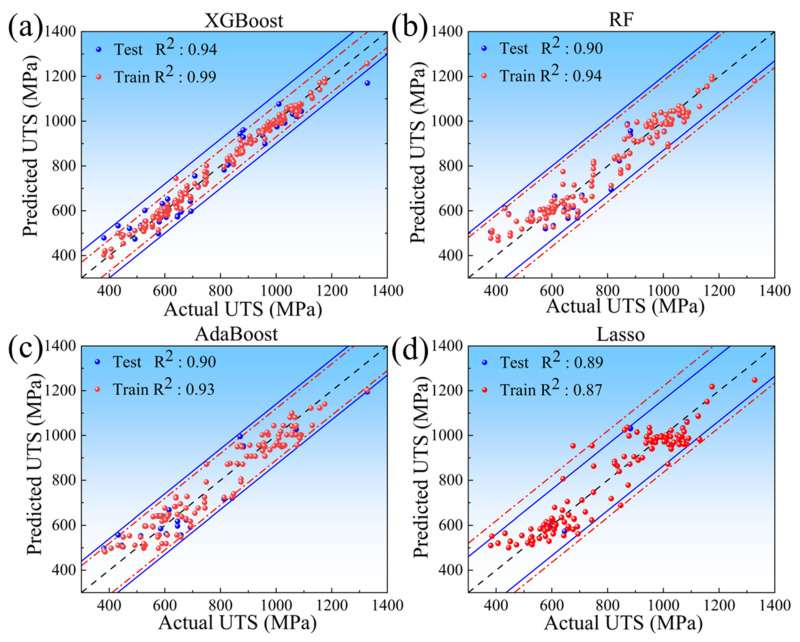
The prediction results of the UTS by four ML models: (**a**) XGBoost; (**b**) RF; (**c**) Adaboost; (**d**) Lasso. (the black dashed line represents the perfect prediction line, the red dot-dashed line represents the distribution range of the training set, and the blue solid line represents the distribution range of the test set).

**Figure 7:**
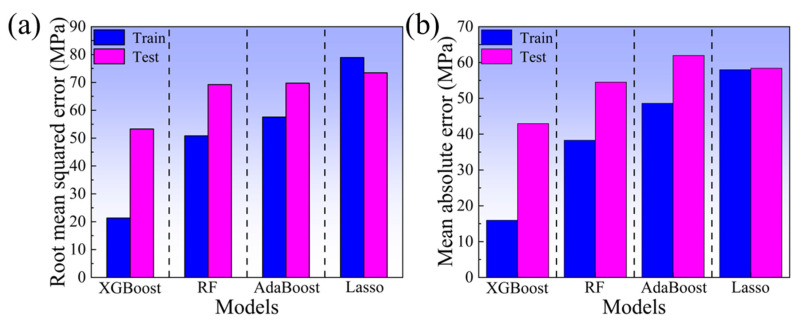
Comparison chart of RMSE and MAE of four ML models: (**a**) RMSE; (**b**) MAE.

The optimal model was determined by comparing the prediction performances of the four ML models on the augmented dataset. Figure 6 shows the prediction results of the UTS by four ML models. The XGBoost model exhibits the most concentrated data distributions, achieving R^2^ values of 0.99 and 0.94 for the training and test sets, respectively, which are substantially higher than those of the other models. Figure 7 shows the RMSE and MAE of four ML models in the task of predicting the UTS. The results indicate that the XGBoost model has the smallest prediction error. The RMSE and MAE values of the training set are 21.32 and 15.96, respectively, while those of the test set are 53.31 and 42.93, respectively. In conclusion, the XGBoost model demonstrates the best prediction performance in the task of predicting the UTS. It is capable of accurately establishing the mapping relationship between the input variables (alloy composition, test temperature) and the output variable (UTS). XGBoost’s exceptional performance stems from integrating a regularization term into its objective function, which controls model complexity and prevents overfitting. Additionally, XGBoost gradually optimizes the objective function through gradient boosting and combines second-order Taylor expansion to accurately fit non-linear relationships, enabling it to perform outstandingly in complex non-linear problems [39,40,41]. Compared with RF, AdaBoost, and Lasso, the XGBoost model is more suitable for predicting the non-linear functional relationship between the UTS of alloys and multi-dimensional independent variables (alloy composition and test temperature). Therefore, the XGBoost model was selected for the design of high-temperature titanium alloy compositions in this study.

### 3.3. Optimal Alloy Composition Design

**Figure 8:**
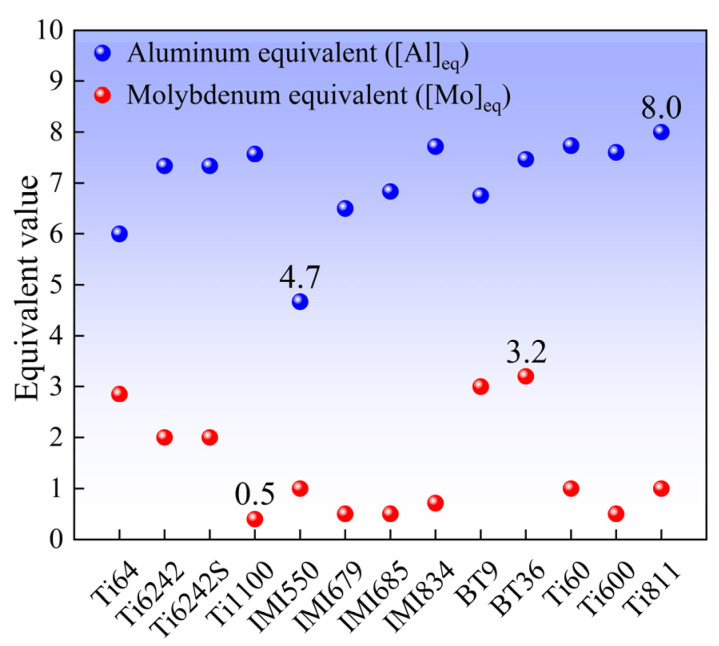
The values of [Al]_eq_ and [Mo]_eq_ of typical high-temperature titanium alloys.

When using ML for alloy composition design, if there are no constraints on the alloy composition, it is possible to generate a large number of solutions that are “high performance based on AI hallucinations” but not feasible in practice. When the content of the Al element in the alloy is relatively high, a large amount of the brittle phase (Ti_3_Al) will precipitate from the α phase under high-temperature service conditions, thus reducing the plasticity of the alloy [42,43]. Meanwhile, elements such as Mo, Nb, and Si are β-stabilizing elements. Excessive addition of these elements will transform the alloy into a β-type titanium alloy, failing to meet the requirements for high-temperature titanium alloys. To address these challenges, this study utilized the physical metallurgical model [Al]_eq_ to constrain the Al content, and the physical metallurgical model [Mo]_eq_ to constrain the Mo and Nb contents, with their expressions shown in Equations (5) and (6) [44,45], respectively. Figure 8 shows the [Al]_eq_ and [Mo]_eq_ of the typical high-temperature titanium alloys, and the results show that the distribution interval of [Al]_eq_ is 4.7~8.0, and the distribution interval of [Mo]_eq_ is 0.5~3.2. Accordingly, the content of Al was limited to 4.7~8.0 wt.%, Mo was 0.5~2.5 wt.%, and Nb was 0~2.3 wt.%. For Si, the content in commonly used high-temperature titanium alloys is 0.1~0.5 wt.% [46]. Next, the genetic algorithm was used to search within the defined compositional range, targeting the UTS at 600 °C. The alloy composition corresponding to the highest UTS was calculated. Table 2 shows the alloy composition and its predicted UTS value at 600 °C.

[Al]_eq_ = Al% + (Sn/3)% + (Zr/6)% + 10(O)% (5)

[Mo]_eq_ = Mo% + (Nb/3.3)% + (Ta/4)% + (W/2)% + (Cr/0.6)% + (Mn/0.6)% + (V/1.4)% + (Fe/0.6)% + (Co/0.9)% + (Ni/0.8)%(6)

### 3.4. Experimental Verification and Analysis

#### 3.4.1. Validation of Prediction Results

**Figure 9:**
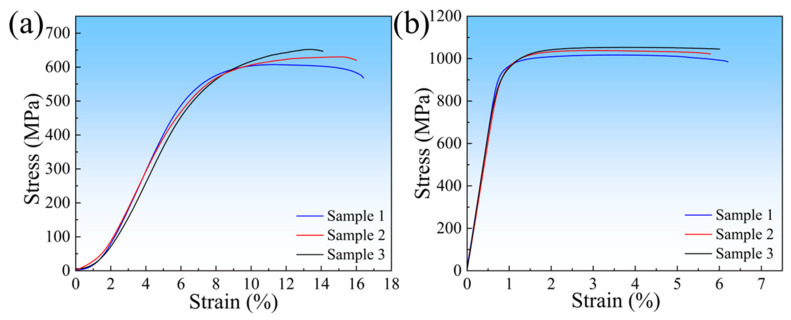
Tensile stress–strain curves of the as-cast alloy: (**a**) 600 °C; (**b**) room temperature.

The tensile stress–strain curves of the alloy at 600 °C and room temperature are shown in Figure 9, and the corresponding mechanical property values are listed in Table 3. As shown in Figure 9 and Table 3, the average values of UTS, YS, and EL of the alloy at 600 °C are 629 MPa (σ = 18.38 MPa), 478 MPa (σ = 14.19 MPa), and 15.5% (σ = 0.97%), respectively. Equations (7) and (8) are used to calculate the experimental error and the prediction error of the XGBoost model. The results show that the experimental error is 2.9%, and the prediction error of the model for the UTS of the alloy at 600 °C is 3.2%. The analysis indicates that the model error and the experimental error are of the same order of magnitude, and both errors are relatively small. On one hand, the small experimental error fully demonstrates the reliability of the experimental data; on the other hand, the small prediction error reflects that the ML-based design method for high-temperature titanium alloys proposed in this study can accurately predict the mechanical properties, verifying the effectiveness of the design scheme. At room temperature, the alloy also exhibits relatively excellent performance, with average values of UTS, YS, and EL being 1035 MPa (σ = 14.76 MPa), 965 MPa (σ = 9.71 MPa), and 5.9% (σ = 0.21%), respectively.

Error_e_ = (σ/UTS_a_) × 100% (7)

Error_p_ = [(UTS_p_ − UTS_a_)/UTS_a_] × 100% (8)

where Error_e_ is the experimental error of the model, σ is the standard deviation, UTS_a_ is the average value of the experimental UTS, Error_p_ is the prediction error of the model, and UTS_p_ is the predicted value of the UTS by the model.

Table 4 presents the UTS data of the novel alloy and two typical as-cast high-temperature titanium alloys (Ti1100 and Ti6242S, both containing six elements). As shown in Table 4, the room-temperature UTS of the novel alloy is higher than that of the two typical high-temperature titanium alloys mentioned above, and its 600 °C high-temperature UTS is higher than that of Ti6242S, but slightly lower than that of Ti1100. The novel alloy has successfully reduced the number of alloying elements while ensuring high performance. This fully demonstrates the excellent potential of ML-assisted design methods in predicting mechanical properties and optimizing compositions within the field of titanium alloy development, and opening up a new path for the efficient development of high-performance and low-cost titanium alloys.

#### 3.4.2. Feature Importance Analysis

**Figure 10:**
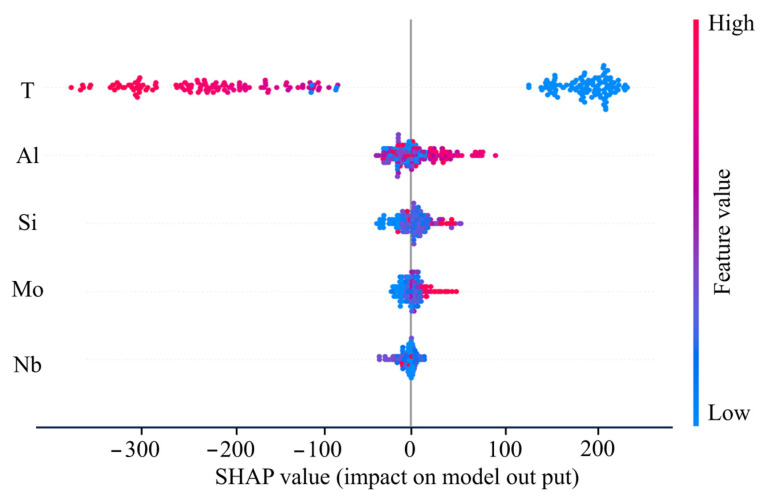
The SHAP analysis results of the XGBoost model.

In the field of ML research, the SHapley Additive exPlanations (SHAP) method transforms the “black-box” decisions of ML models into interpretable feature importance distributions by quantifying the contribution of each feature to the model’s prediction results [48,49]. To understand the influence of input features (test temperature, alloy composition) on the UTS, this study used the SHAP method to analyze the feature importance of the XGBoost model. Figure 10 shows the SHAP analysis results of the XGBoost model, where red indicates higher feature values (composition content and test temperature), and blue indicates lower feature values. The horizontal axis represents the SHAP value, whose magnitude reflects the degree to which the feature affects the UTS. As shown in Figure 10, the test temperature is the most important feature affecting the UTS. SHAP values decrease with increasing test temperatures, indicating that the UTS of the alloy decreases with the increase in the test temperature. The feature importance ranking of alloy compositions is Al > Si > Mo > Nb, suggesting that Al is a key feature influencing the UTS. A higher Al content (red) corresponds to a more positive SHAP value, indicating that an increase in Al content is beneficial to improving the UTS. A similar trend was observed for Mo content. The SHAP values of Si and Nb do not show obvious patterns with their composition contents, and their contributions to the alloy’s UTS are complex, suggesting that the effects of Si and Nb elements on the UTS of the alloy are largely influenced by other elements.

#### 3.4.3. Microstructure Characterization

**Figure 11:**
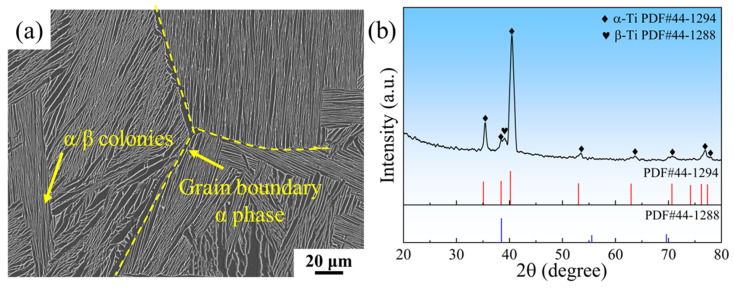
The microstructure and XRD diffraction pattern of the alloy: (**a**) SEM morphology; (**b**) XRD diffraction pattern.

Figure 11 shows the microstructure and the XRD diffraction pattern of the alloy. The microstructure of the alloy is a Widmanstätten structure, which is a typical microstructure of high-temperature titanium alloys. There is an obvious grain boundary α phase at the grain boundaries. At the same time, α/β bundles in different directions exist within the grains. This is because the α phase in the β grains needs to follow the 12 Burgers orientation relationships: (0001)_α_//(110)_β_ and <11$\bar{2}$0>_α_//<111>_β_ [50]. The quantitative statistical analysis of the microstructural characteristics was conducted using Image-Pro Plus software (V6.0, Media Cybernetics, Rockville, MD, USA) (average values calculated from measurements of 10 SEM images). The results show that the thickness of α-laths is 0.85 μm, the size of α/β colonies is 26.0 μm, and the volume fraction of the α phase is 75.4%. The analysis of the XRD diffraction pattern of the alloy shows that the phase of the alloy consists of an α-phase and a small amount of β-phase, classifying the designed alloy as an α+β type titanium alloy.

#### 3.4.4. Fracture Mechanism Analysis

**Figure 12:**
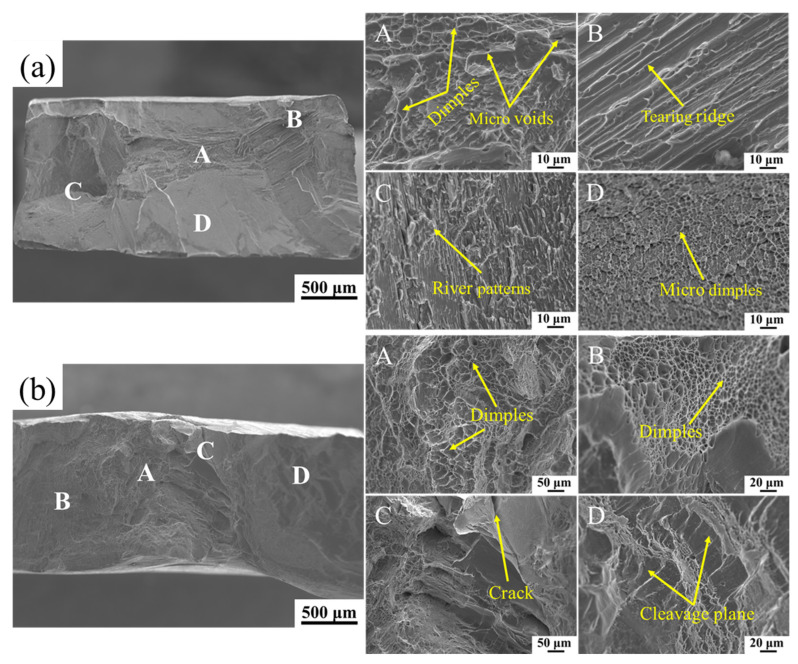
SEM morphology of tensile fracture surfaces of the alloy: (**a**) room temperature; (**b**) 600 °C.

Figure 12a shows the SEM morphology of the tensile fracture surface of the alloy at room temperature. Region A exhibits remarkable dimple characteristics, indicating the occurrence of ductile trans-granular fracture. Regions B and C are characterized by typical tearing ridge and river patterns, respectively, suggesting a brittle trans-granular fracture in these two regions. However, Region D exhibits a bright and reflective surface at the macroscopic level, and has the characteristic of tiny dimples at the microscopic level, indicating the occurrence of ductile intergranular fracture. Therefore, the fracture mode of the alloy at room temperature is a mixed fracture of brittleness and ductility. Figure 12b shows the SEM morphology of the tensile fracture surface of the alloy at 600 °C. Regions A and B exhibit the characteristics of dimples, pointing to ductile trans-granular fracture in these two regions. A crack is observed in Region C, while Region D shows the characteristics of cleavage plane, indicating that brittle trans-granular fracture occurred in these two regions. Therefore, the high-temperature fracture mode of the alloy is also a mixed fracture of brittleness and ductility.

The room-temperature fracture mechanism of the alloy is interpreted as follows: As the specimen deforms under the tensile load, the central position (Region A) elongates perpendicular to the tensile axis, thereby forming dimples and tiny pores. Driven by cumulative effect and stress concentration, these pores gradually propagate to the adjacent region, resulting in brittle trans-granular fracture in Region B and Region C. When the center of the specimen is completely separated, this failure will spread outward. Due to the change in the elastoplastic constraint, the maximum shear plane (at a 45° angle to the tensile axis) is likely to become the deformation plane, inducing ductile intergranular fracture at the grain boundaries (Region D). The high-temperature fracture mechanism of the alloy is interpreted as follows: The central position initially elongates perpendicular to the tensile axis. With the increase in the tensile temperature and the plastic strain reaching a critical level, obvious plastic deformation occurs, forming a large number of deep dimples in Region A. With the continued deformation, due to the material-softening effect caused by the high temperature, a ductile trans-granular fracture occurs in Region B (located on the side of the alloy), accompanied by pronounced shear fracture characteristics, resulting in a typical dimple morphology. The formation of the crack in region C arises from a large amount of deformation-induced significant inhomogeneous plastic deformation at high temperatures, triggering the initiation of the crack and the brittle trans-granular fracture in Region D.

## 4. Conclusions

This study successfully employed an ML method to design a novel high-temperature titanium alloy, Ti-7.2Al-1.8Mo-2.0Nb-0.4Si (wt.%), and validated its feasibility. The specific conclusions are as follows:

(1) Data augmentation significantly improved the performance of all four ML models. This indicated that data augmentation enabled the models to better learn the dataset information more effectively, thereby enhancing the generalization ability and prediction accuracy of the models.

(2) By training and testing the augmented dataset, the XGBoost model exhibited superior prediction performance with the R^2^ of 0.94, RMSE of 53.31, and MAE of 42.93 for the test set.

(3) Combining the physical metallurgy model with the genetic algorithm improved the efficiency and accuracy of alloy design. The UTS, YS, and EL of the designed alloy at 600 °C were 629 MPa, 478 MPa, and 15.5%, respectively. The predicted UTS of the alloy at 600 °C by the XGBoost model was 649 MPa, with a minimal error of only 3.2%, which verified the efficacy of the machine learning method used in this study for designing novel high-temperature titanium alloys. At room temperature, the UTS, YS, and EL of the alloy were 1035 MPa, 965 MPa, and 5.9%, respectively.

(4) The microstructure analysis revealed that the designed high-temperature titanium alloy was of the α+β type, and its structure was a typical Widmanstätten structure. The fracture modes of the alloy at room temperature and high temperature were mixed fractures of brittleness and ductility.

This study has achieved remarkable results in the field of composition design for a novel quinary high-performance high-temperature titanium alloy through machine learning methods. Compared to as-cast Ti1100 and Ti6242S alloy (both containing six elements), the novel alloy has considerable high-temperature (600 °C) mechanical properties and fewer components. In the future, the continuous optimization of ML approaches will continue to drive the discovery and design of high-performance materials, providing a technical pathway that balances efficiency and reliability for the precise design of advanced structural materials.

## Figures and Tables

**Table 1:** Dataset information.

Variable		Unit	Data Range	
Input	Composition	wt.%	Al	3.2~10.0
			Sn	0~6.2
			Zr	0~11.0
			Mo	0~4.05
			Si	0~1.5
			Nb	0~6.5
			Ta	0~4.0
			W	0~3.6
			Y	0~0.62
			V	0~4.5
	Testing temperature	°C	T	20~750
Output	Ultimate tensile strength	MPa	UTS	381~1328

**Table 2:** Alloy composition and prediction of UTS at 600 °C.

Alloy	Test Temperature (°C)	UTS (MPa)
Ti-7.2Al-1.8Mo-2.0Nb-0.4Si	600	649

**Table 3:** The mechanical properties of as-cast alloy at room temperature and 600 °C.

	600 °C			Room Temperature		
	UTS (MPa)	YS (MPa)	EL (%)	UTS (MPa)	YS (MPa)	EL (%)
Sample 1	607	465	16.3	1016	954	6.2
Sample 2	628	472	16.0	1037	968	5.7
Sample 3	652	498	14.1	1052	975	6.0
Average value	629	478	15.5	1035	965	5.9
Standard deviation σ	18.38	14.19	0.97	14.76	9.71	0.21

**Table 4:** Comparison of UTS with two typical as-cast high-temperature titanium alloys.

Alloy	Test Temperature (°C)	UTS (MPa)
Ti-6Al-2.7Sn-4Zr-0.4Mo-0.45Si (Ti1100) [47]	20	930
	600	650
Ti-6Al-2Sn-4Zr-2Mo-0.1Si (Ti6242S) [47]	20	1020
	600	595
This work	20	1035
	600	629

## Data Availability

The original contributions presented in this study are included in this article; further inquiries can be directed to the corresponding author.

## Supplementary Materials

The following supporting information can be downloaded at: https://www.mdpi.com/article/10.3390/ma18133099/s1, Table S1: Original data; Table S2: Augmented data.
