# Supplementary material for: Composition Design of a Novel High-Temperature Titanium Alloy Based on Data Augmentation Machine Learning
Source: Materials (Basel). 2025 Jun 30;18(13):3099. doi: 10.3390/ma18133099 (PMC12250823; doi:10.3390/ma18133099)
Supplement: Supplementary file 1 [file materials-18-03099-s001.zip › materials-3659413-supplementary.pdf]

Table S1: Original data

Al (wt.%)	Sn (wt.%)	Zr (wt.%)	Mo (wt.%)	Si (wt.%)	Nb (wt.%)	Ta (wt.%)	W (wt.%)	Y (wt.%)	V (wt.%)	T (℃)	UTS (MPa)
5.61	3.69	3.45	0.99	0.38	0.4	0.2	0	0	0	20	940
5.91	4.07	3.92	0.51	0.27	0.82	0	0	0	0	550	645
5.92	2.12	3.88	2.03	0.1	0	0	0	0	0	600	571
5.41	2.52	5.33	0.68	0.19	1.35	1.33	1.35	0.62	0	650	603
6.52	4.25	5.5	2.73	0.4	1.77	0	3.58	0.1	0	700	661
6.5	0	1.5	3.5	0.3	0	0	0	0	0	20	1075
6.5	0	1.5	3.5	0.3	0	0	0	0	0	500	656
6.64	1.89	3.73	4.05	0.2	0	0	1.01	0	0	20	1085
6.64	1.89	3.73	4.05	0.2	0	0	1.01	0	0	550	813
5.4	6.2	6.4	0	0.5	0	4	1.6	0	0	20	1328
5.4	6.2	6.4	0	0.5	0	4	1.6	0	0	600	1017
5.4	6.2	6.4	0	0.5	0	4	1.6	0	0	650	842
5.4	6.2	6.4	0	0.5	0	4	1.6	0	0	700	640
5.4	6.2	0.5	0	0.5	0	0	0	0	0	20	1003
5.4	6.2	0.5	0	0.5	0	0	0	0	0	600	610
5.4	6.2	0.5	0	0.5	0	0	0	0	0	650	432
6	0	0	0	0	0	0	0	0.01	4	20	925
8.7	0	0	0.5	0	0	0	0	0	0.5	20	750
6	0	0	0	0	0	0	0	0	4.5	20	895

6.85	0	0	0	0	0	0	0	0	3.6	20	910
8.69	0	0	0.72	0	0	0	0	0	0.78	20	826
8.35	0	0	0.9	0	0	0	0	0	1.03	20	825
8.7	0	0	0.76	0	0	0	0	0	0.78	20	825
9.53	0	0	0.75	0	0	0	0	0	1.43	20	880
6.52	4.25	5.5	2.73	0.4	1.77	0	3.58	0.1	0	20	1125
6.52	4.25	5.5	2.73	0.4	1.77	0	3.58	0.1	0	750	605
6.59	4.24	5.5	2.72	0.43	1.71	0.98	3.6	0.11	0	20	1157
6.59	4.24	5.5	2.72	0.43	1.71	0.98	3.6	0.11	0	700	708
6.59	4.24	5.5	2.72	0.43	1.71	0.98	3.6	0.11	0	750	610
6.5	4.2	5.55	2.77	0.42	1.78	1.99	3.59	0.09	0	20	1175
6.5	4.2	5.55	2.77	0.42	1.78	1.99	3.59	0.09	0	700	874
6.5	4.2	5.55	2.77	0.42	1.78	1.99	3.59	0.09	0	750	749
4.543	2.567	2.134	1.403	0.419	2.048	0	1.089	0.129	0	20	1011.6
4.543	2.567	2.134	1.403	0.419	2.048	0	1.089	0.129	0	700	693.9
4.543	2.567	2.134	1.403	0.419	2.048	0	1.089	0.129	0	750	531.8
4.671	2.885	2.849	1.465	0.461	2.074	0	1.067	0.277	0	20	905.5
4.671	2.885	2.849	1.465	0.461	2.074	0	1.067	0.277	0	700	645.7
4.671	2.885	2.849	1.465	0.461	2.074	0	1.067	0.277	0	750	555.6
4.578	2.898	2.867	1.479	0.517	2.072	0	1.144	0.388	0	20	1028
4.578	2.898	2.867	1.479	0.517	2.072	0	1.144	0.388	0	700	676.4

4.578	2.898	2.867	1.479	0.517	2.072	0	1.144	0.388	0	750	528.3
4.598	2.73	5.787	0.771	0.436	0.676	0	1.084	0.139	0	20	1059.1
4.598	2.73	5.787	0.771	0.436	0.676	0	1.084	0.139	0	700	643.1
4.598	2.73	5.787	0.771	0.436	0.676	0	1.084	0.139	0	750	513.3
4.543	2.905	5.643	0.784	0.325	1.279	0	1.05	0.128	0	20	1090.7
4.543	2.905	5.643	0.784	0.325	1.279	0	1.05	0.128	0	70	744.3
4.543	2.905	5.643	0.784	0.325	1.279	0	1.05	0.128	0	750	529.3
4.743	2.744	5.709	0.799	0.326	2.113	0	1.202	0.131	0	20	967.7
4.743	2.744	5.709	0.799	0.326	2.113	0	1.202	0.131	0	70	676.1
4.743	2.744	5.709	0.799	0.326	2.113	0	1.202	0.131	0	750	473.2
6	3.75	4	0.4	0.45	0.5	0	0	0	0	20	972
6	3.75	4	0.4	0.45	0.5	0	0	0	0	600	591
6	2.75	4	0.4	0.45	1	0	0	0	0	20	1020
6	2.75	4	0.4	0.45	1	0	0	0	0	600	558
6	2.75	4	0.4	0.45	1.5	0	0	0	0	20	1073
6	2.75	4	0.4	0.45	1.5	0	0	0	0	600	585
6	2.75	4	0.4	0.45	2	0	0	0	0	20	1084
6	2.75	4	0.4	0.45	2	0	0	0	0	600	589
4.547	1.873	4.998	0.771	0.303	1.04	0	0.969	0	0	20	1038.45
5.432	2.401	5.402	0.65	0.258	0.851	0	0.797	0	0	20	1057.49
5.089	2.714	7.226	0.575	0.299	0.875	0	0.839	0	0	20	1072.32

6.542	4.445	9.791	0.6	0.218	0.868	0	0.848	0	0	20	1056.55
6	4	7	0.8	0.25	1	0	1	0	0	20	1130
6	4	7	0.8	0.25	1	0	1	0	0	650	575
6	4	7	0.8	0.25	1	0	1	0	0	700	524
6	4	7	0.8	0.25	1	0	1	0	0	750	381
6	4	9	0.8	0.25	1	0	1	0	0	20	1068
6	4	9	0.8	0.25	1	0	1	0	0	650	658
6	4	9	0.8	0.25	1	0	1	0	0	700	577
6	4	9	0.8	0.25	1	0	1	0	0	750	407
6	4	11	0.8	0.25	1	0	1	0	0	20	1050
6	4	11	0.8	0.25	1	0	1	0	0	650	692
6	4	11	0.8	0.25	1	0	1	0	0	700	605
6	4	11	0.8	0.25	1	0	1	0	0	750	451
5.41	2.52	5.33	0.68	0.19	1.35	1.33	1.35	0.62	0	20	1070
6.5	0	1.5	3.5	0.3	0	0	0	0	0	650	590
6	2	4	1	0.5	0	0	0	0	0	20	1010
6	2	4	1	0.5	0	0	0	0	0	550	635
6	2	4	1	0.5	0	0	0	0	0	600	615
5.8	4.2	3.9	0	0.4	0.7	1.5	0	0	0	20	871
6	3	10	1	0.25	1	0	1	0	0	20	1026.2
6	3	10	1	0.25	1	0	1	0	0	700	492.5

6.56	2.36	4.36	2.3	0.14	0	0	0	0	0	20	1010
5.92	2.12	3.88	2.03	0.1	0	0	0	0	0	20	955
5.92	2.12	3.88	2.03	0.1	0	0	0	0	0	500	638
5.41	1.8	3.45	1.8	0.07	0	0	0	0	0	20	938
5.91	4.07	3.92	0.51	0.27	0.82	0	0	0	0	20	1031
5.91	4.07	3.92	0.51	0.27	0.82	0	0	0	0	600	609
7.5	0	2	0	1.5	2	0	0	0	2	20	861
7.8	0	2.2	0	0.8	0	0	0	0	1	20	958
7.8	0	2.2	0	0.8	0	0	0	0	1	500	680
7.8	0	2.2	0	1.4	0	0	0	0	1	20	993
7.8	0	2.2	0	1.4	0	0	0	0	1	500	847
7.8	0	2.2	0	1.4	0	0	0	0	1	600	743
8	0	2.2	0	0.8	0	0	0	0	1	20	1028
8	0	2.2	0	1.4	0	0	0	0	1	20	1020
8	0	2	0	1.5	1	0	0	0	0	20	877
8	0	2	1	1.5	0	0	0	0	0	20	882
8.5	0	2	0	1.5	1	0	0	0.15	0	20	978
8.5	0	2	0	1.5	0	0.2	0	0.15	1	20	952
10	0	0	1	0	0	0	0	0	0	20	835
9.3	0	0	2	0	0	0	0	0	0	20	860
3.2	0	0	0.5	0	6.5	0.5	0	0	0.5	20	960

3.2	0	0	0.5	0	6.5	0.5	0	0	0.5	500	600
3.2	0	0	0.5	0	6.5	0.5	0	0	0.5	700	445
5	0	1.32	0.45	0	6.5	1.32	0	0	0.22	20	960
5	0	1.32	0.45	0	6.5	1.32	0	0	0.22	500	720
5	0	1.32	0.45	0	6.5	1.32	0	0	0.22	600	660
5	0	1.32	0.45	0	6.5	1.32	0	0	0.22	700	590
5.8	4	4	0	0.4	0.7	1.5	0	0	0	20	950
6	3	10	0.8	0.3	1	0	1	0	0	600	580
6	3	10	0.8	0.3	1	0	1	0	0	650	525
6	3	10	0.8	0.3	1	0	1	0	0	700	385
6	3	10	0.8	0.3	1	0	1	0	0	20	992

Table S2: Augmented data

Al (wt.%)	Sn (wt.%)	Zr (wt.%)	Mo (wt.%)	Si (wt.%)	Nb (wt.%)	Ta (wt.%)	W (wt.%)	Y (wt.%)	V (wt.%)	T (℃)	UTS (MPa)
5.616385925	3.673384799	3.449339202	0.977889838	0.373481639	0.400473987	0.191395866	0	0	0	19.99615444	940
5.926690215	4.067404086	3.90496857	0.507542569	0.267272764	0.793031134	0	0	0	0	549.9994571	645
5.938820245	2.1334542	3.895931866	2.024887843	0.090103952	0	0	0	0	0	599.9987421	571
5.413711459	2.513960148	5.330865898	0.678443228	0.201677821	1.352544208	1.333376027	1.34588123	0.615123938	0	649.9956744	603
6.538861859	4.251745778	5.502575504	2.729255541	0.380812288	1.769734861	0	3.580602302	0.124632421	0	699.9980764	661
6.5	4.2	5.55	2.77	0.42	1.78	1.99	3.59	0.09	0	20	1175
6.503944521	0	1.495790155	3.502897749	0.320754008	0	0	0	0	0	650.0087112	590
6.52	4.25	5.5	2.73	0.4	1.77	0	3.58	0.1	0	750	605
4.585621845	2.751330334	5.767479122	0.769482149	0.441883172	0.678809919	0	1.077773005	0.136918777	0	749.99507	513.3
9.3	0	0	2	0	0	0	0	0	0	20	860
6.523015473	4.249652882	5.48831322	2.741428228	0.40751933	1.777910319	0	3.570906125	0.114027943	0	749.9859815	605
4.743	2.744	5.709	0.799	0.326	2.113	0	1.202	0.131	0	20	967.7
6.52	4.25	5.5	2.73	0.4	1.77	0	3.58	0.1	0	700	661
6.5	0	1.5	3.5	0.3	0	0	0	0	0	20	1075
5.41	1.8	3.45	1.8	0.07	0	0	0	0	0	20	938
5.991243817	3.986172003	11.00926178	0.819094166	0.236014324	1.005629692	0	0.993493574	0	0	19.99512875	1050
6	4	9	0.8	0.25	1	0	1	0	0	650	658
5.8	4.2	3.9	0	0.4	0.7	1.5	0	0	0	20	871
4.540823188	2.915987769	5.651254163	0.792135096	0.338054788	1.279210038	0	1.05681953	0.124897332	0	750.0032417	529.3

6.000125924	2.750976761	3.992269902	0.400245102	0.454979983	1.014511436	0	0	0	0	600.0095927	558
3.20982691	0	0	0.516654744	0	6.510143701	0.481591258	0	0	0.48720423	699.9937518	445
5.983935537	4.002034636	8.992436493	0.785777463	0.243534271	0.98918452	0	1.016871416	0	0	700.0088164	577
6.008339222	3.004591801	9.999298343	0.783390391	0.304296182	1.002076877	0	1.002715788	0	0	649.9872325	525
6.592598828	4.247818229	5.487630493	2.706795434	0.435219416	1.712969847	0.982504929	3.603464482	0.103199753	0	750.0023225	610
4.694146586	2.866327348	2.855862602	1.448872841	0.456280681	2.084889506	0	1.0676428	0.266222552	0	699.992847	645.7
6	2.75	4	0.4	0.45	1	0	0	0	0	600	558
8.5	0	2	0	1.5	0	0.2	0	0.15	1	20	952
7.794531411	0	2.199672467	0	0.794565752	0	0	0	0	0.992871542	20.0010643	958
6.009504238	2.744230963	3.991015853	0.404919192	0.436797668	2.018314588	0	0	0	0	20.0117944	1084
5.926543657	2.119444153	3.882799686	2.01874511	0.12445752	0	0	0	0	0	20.00129221	955
8.693571126	0	0	0.73477894	0	0	0	0	0	0.774817298	19.99191506	826
4.578	2.898	2.867	1.479	0.517	2.072	0	1.144	0.388	0	20	1028
6.59	4.24	5.5	2.72	0.43	1.71	0.98	3.6	0.11	0	700	708
5.91	4.07	3.92	0.51	0.27	0.82	0	0	0	0	20	1031
4.749241198	2.750283455	5.708877532	0.790027456	0.326758046	2.106228383	0	1.211751197	0.129529426	0	69.99174503	676.1
8.010490092	0	1.992956563	0.985915387	1.484433708	0	0	0	0	0	20.0060601	882
5.4	6.2	6.4	0	0.5	0	4	1.6	0	0	700	640
8.35	0	0	0.9	0	0	0	0	0	1.03	20	825
8.715380366	0	0	0.49964174	0	0	0	0	0	0.515646437	19.97380255	750
5.61	3.69	3.45	0.99	0.38	0.4	0.2	0	0	0	20	940

6.564134349	2.378767958	4.352262108	2.287553453	0.122212798	0	0	0	0	0	20.01496044	1010
8.00326133	0	1.987488864	0	1.50924027	0.998150979	0	0	0	0	19.99477277	877
6	0	0	0	0	0	0	0	0.01	4	20	925
6	3	10	0.8	0.3	1	0	1	0	0	20	992
6	4	9	0.8	0.25	1	0	1	0	0	750	407
6.5	0	1.5	3.5	0.3	0	0	0	0	0	500	656
6.522610553	4.250051135	5.497654129	2.715846293	0.395793547	1.766572855	0	3.571977227	0.098387143	0	20.00404051	1125
6.504967142	0	1.498617357	3.506476885	0.315230299	0	0	0	0	0	19.99765847	1075
4.585522168	2.746324113	5.772698586	0.766599555	0.437307406	0.690412733	0	1.069641378	0.150631638	0	20.00010233	1059.1
5.994006074	2.728761043	3.99474245	0.392408673	0.451503938	1.50341756	0	0	0	0	600.0187617	585
4.543	2.567	2.134	1.403	0.419	2.048	0	1.089	0.129	0	700	693.9
5.996739765	2.012012139	3.995919246	0.979618755	0.489919137	0	0	0	0	0	19.98129208	1010
4.671	2.885	2.849	1.465	0.461	2.074	0	1.067	0.277	0	20	905.5
6.008303358	2.991439162	10.00071566	0.995223426	0.254789798	1.003336621	0	1.010375399	0	0	19.99489984	1026.2
6	3	10	1	0.25	1	0	1	0	0	700	492.5
6.59	4.24	5.5	2.72	0.43	1.71	0.98	3.6	0.11	0	20	1157
4.543	2.567	2.134	1.403	0.419	2.048	0	1.089	0.129	0	750	531.8
6	2.75	4	0.4	0.45	1.5	0	0	0	0	20	1073
4.543	2.905	5.643	0.784	0.325	1.279	0	1.05	0.128	0	750	529.3
8	0	2.2	0	0.8	0	0	0	0	1	20	1028
5.4	6.2	6.4	0	0.5	0	4	1.6	0	0	650	842

8	0	2	0	1.5	1	0	0	0	0	20	877
6.5	4.2	5.55	2.77	0.42	1.78	1.99	3.59	0.09	0	700	874
4.578	2.898	2.867	1.479	0.517	2.072	0	1.144	0.388	0	700	676.4
9.999430544	0	0	1.003078018	0	0	0	0	0	0	19.98289832	835
6	2.75	4	0.4	0.45	2	0	0	0	0	20	1084
3.2	0	0	0.5	0	6.5	0.5	0	0	0.5	500	600
5.92	2.12	3.88	2.03	0.1	0	0	0	0	0	500	638
6.850917608	0	0	0	0	0	0	0	0	3.580124311	19.99780328	910
4.543	2.905	5.643	0.784	0.325	1.279	0	1.05	0.128	0	20	1090.7
6.595868571	4.261904556	5.490094637	2.714337023	0.430996514	1.704965243	0.964493366	3.60068563	0.099376963	0	20.00473592	1157
7.8	0	2.2	0	0.8	0	0	0	0	1	500	680
6.532350765	4.451860515	9.801584245	0.582412605	0.206167415	0.847607678	0	0.845305932	0	0	20.00717542	1056.55
6	2	4	1	0.5	0	0	0	0	0	20	1010
9.53	0	0	0.75	0	0	0	0	0	1.43	20	880
5.979325579	3.9991088	6.986955305	0.806696725	0.253665982	0.990601202	0	0.994861331	0	0	649.9894079	575
4.671	2.885	2.849	1.465	0.461	2.074	0	1.067	0.277	0	700	645.7
5	0	1.32	0.45	0	6.5	1.32	0	0	0.22	700	590
8.705132674	0	0	0.760970775	0	0	0	0	0	0.78968645	19.99297947	825
4.571466708	2.915654542	2.871049817	1.46639116	0.526178619	2.093221562	0	1.154324653	0.3728063	0	699.9951577	676.4
6	3	10	0.8	0.3	1	0	1	0	0	650	525
6.542	4.445	9.791	0.6	0.218	0.868	0	0.848	0	0	20	1056.55

8.34498243	0	0	0.909154021	0	0	0	0	0	1.033287511	19.9947024	825
4.743	2.744	5.709	0.799	0.326	2.113	0	1.202	0.131	0	70	676.1
6.643142473	1.880919759	3.715876963	4.064656488	0.197742237	0	0	1.010675282	0	0	549.9857525	813
6.56	2.36	4.36	2.3	0.14	0	0	0	0	0	20	1010
5.91	4.07	3.92	0.51	0.27	0.82	0	0	0	0	550	645
6	4	11	0.8	0.25	1	0	1	0	0	700	605
6.5	4.2	5.55	2.77	0.42	1.78	1.99	3.59	0.09	0	750	749
7.797450228	0	2.21503993	0	0.773490302	0	0	0	0	1.010915069	500.0124609	680
7.8	0	2.2	0	1.4	0	0	0	0	1	600	743
6	4	7	0.8	0.25	1	0	1	0	0	650	575
4.547052437	1.873469806	4.993499345	0.777228499	0.292323796	1.038576205	0	0.970202956	0	0	20.00514439	1038.45
6	4	11	0.8	0.25	1	0	1	0	0	750	451
7.779266098	0	2.196573124	0	1.396285591	0	0	0	0	0.985924883	19.99222183	993
7.8	0	2.2	0	0.8	0	0	0	0	1	20	958
6	3.75	4	0.4	0.45	0.5	0	0	0	0	600	591
5.410557249	1.810941915	3.433075354	1.815295503	0.068419921	0	0	0	0	0	19.99573119	938
4.537106352	2.913496021	5.646570155	0.777070904	0.333995999	1.282072995	0	1.058128621	0.134296288	0	19.99171005	1090.7
4.598	2.73	5.787	0.771	0.436	0.676	0	1.084	0.139	0	20	1059.1
5	0	1.32	0.45	0	6.5	1.32	0	0	0.22	20	960
6.005193465	2.765327389	3.998912399	0.404017117	0.45690144	0.995987795	0	0	0	0	20.00224092	1020
4.571382135	2.906524333	2.859074793	1.477852636	0.522049873	2.080657552	0	1.131997036	0.384654988	0	19.99525055	1028

4.53739819	2.912472936	5.649103703	0.783790984	0.326173274	1.291776649	0	1.044084286	0.133470974	0	69.99797807	744.3
5.394556173	6.201109226	6.388490064	0	0.50375698	0	3.993993613	1.597083063	0	0	19.99398293	1328
7.788894242	0	2.217522704	0	1.409356784	0	0	0	0	1.012715551	500.0072167	847
5.001848361	0	1.311416422	0.457003099	0	6.494243622	1.321220098	0	0	0.245600845	599.9990394	660
4.598	2.73	5.787	0.771	0.436	0.676	0	1.084	0.139	0	750	513.3
6	3	10	1	0.25	1	0	1	0	0	20	1026.2
6	3	10	0.8	0.3	1	0	1	0	0	700	385
4.543	2.905	5.643	0.784	0.325	1.279	0	1.05	0.128	0	70	744.3
6	2	4	1	0.5	0	0	0	0	0	550	635
6.008219025	0	0	0	0	0	0	0	0	4.500870471	19.99700993	895
6.59	4.24	5.5	2.72	0.43	1.71	0.98	3.6	0.11	0	750	610
6.49765863	0	1.515792128	3.507674347	0.295305256	0	0	0	0	0	500.0054256	656
6.580805758	4.255499344	5.492167467	2.716779385	0.438135172	1.697691357	0.982274599	3.613071428	0.093925168	0	700.0018463	708
5.899878956	4.053451433	3.928231706	0.51073318	0.257100391	0.807049212	0	0	0	0	19.99664215	1031
7.8	0	2.2	0	1.4	0	0	0	0	1	20	993
6	4	11	0.8	0.25	1	0	1	0	0	20	1050
5.432	2.401	5.402	0.65	0.258	0.851	0	0.797	0	0	20	1057.49
3.2	0	0	0.5	0	6.5	0.5	0	0	0.5	700	445
5.998400615	3.749809838	3.989974706	0.399814869	0.447113414	0.503227186	0	0	0	0	599.9917277	591
8	0	2	1	1.5	0	0	0	0	0	20	882
10	0	0	1	0	0	0	0	0	0	20	835

6	0	0	0	0	0	0	0	0	4.5	20	895
5.999920274	4.014799441	9.000773683	0.791387158	0.265231241	1.0053891	0	0.989627538	0	0	749.9980966	407
5.999373209	4.009551423	6.99014274	0.805040465	0.244697424	0.992071272	0	0.998929696	0	0	699.9896476	524
7.5	0	2	0	1.5	2	0	0	0	2	20	861
6	3	10	0.8	0.3	1	0	1	0	0	600	580
5.38893665	6.188037934	0.508125258	0	0.5135624	0	0	0	0	0	649.9992799	432
4.530542612	2.568731809	2.137853174	1.394161426	0.420537251	2.048582087	0	1.077570297	0.132577874	0	700.0056078	693.9
7.788709482	0	2.194754797	0	1.404893746	0	0	0	0	0.987778722	600.00713	743
4.553830512	2.577538021	2.120223306	1.39362175	0.424150353	2.05313786	0	1.094150477	0.167527315	0	750.0057089	531.8
3.190216272	0	0	0.504082528	0	6.482974164	0.510291556	0	0	0.504725975	500.0025603	600
5.92	2.12	3.88	2.03	0.1	0	0	0	0	0	20	955
7.500592184	0	2.000139293	0	1.499758749	2.001980848	0	0	0	1.998556396	19.99426338	861
5.995308243	2.732868655	4.013538724	0.398854602	0.462378163	1.984055723	0	0	0	0	599.9940062	589
5.392801558	6.195393612	6.410571222	0	0.503436183	0	3.982369598	1.60324084	0	0	699.9961492	640
6.52	4.25	5.5	2.73	0.4	1.77	0	3.58	0.1	0	20	1125
8.5	0	2	0	1.5	1	0	0	0.15	0	20	978
5.4	6.2	0.5	0	0.5	0	0	0	0	0	20	1003
6	3.75	4	0.4	0.45	0.5	0	0	0	0	20	972
4.590669111	2.890923305	2.871438194	1.486746341	0.507730695	2.071404746	0	1.111587327	0.377756124	0	749.9974743	528.3
5.994232287	4.007553912	11.00500917	0.790224448	0.250993323	1.007513871	0	0.983305947	0	0	700.0054336	605
5.4	6.2	6.4	0	0.5	0	4	1.6	0	0	20	1328

5.4	6.2	6.4	0	0.5	0	4	1.6	0	0	600	1017
5.994076061	3.991360092	11.00048522	0.791690499	0.252704568	0.999497619	0	0.99761052	0	0	649.9909244	692
5.921093948	2.127257666	3.884810092	2.03223884	0.092095255	0	0	0	0	0	500.0047147	638
5.089	2.714	7.226	0.575	0.299	0.875	0	0.839	0	0	20	1072.32
5	0	1.32	0.45	0	6.5	1.32	0	0	0.22	500	720
6.5	0	1.5	3.5	0.3	0	0	0	0	0	650	590
5.8	4	4	0	0.4	0.7	1.5	0	0	0	20	950
6.635365823	1.885342702	3.732419623	4.030867198	0.182750822	0	0	1.004377125	0	0	19.98987169	1085
6	2.75	4	0.4	0.45	2	0	0	0	0	600	589
6.021531825	2.742326524	4.008723206	0.40183342	0.471898029	1.491917017	0	0	0	0	19.99160278	1073
5.011492733	0	1.312968236	0.449650115	0	6.517708006	1.313730329	0	0	0.238124486	700.0070775	590
4.677795977	2.877696334	2.851164586	1.465455718	0.454483997	2.095439441	0	1.07333919	0.256748574	0	750.0018645	555.6
5.41	2.52	5.33	0.68	0.19	1.35	1.33	1.35	0.62	0	20	1070
6	4	7	0.8	0.25	1	0	1	0	0	20	1130
5.794375332	4.006324077	4.009725544	0	0.4062181	0.684297753	1.492728628	0	0	0	19.99752481	950
6	2.75	4	0.4	0.45	1.5	0	0	0	0	600	585
5.420062928	2.514231081	5.338356921	0.668702931	0.195298042	1.364415686	1.305283555	1.342031047	0.625770721	0	19.99796955	1070
6	2	4	1	0.5	0	0	0	0	0	600	615
5.4	6.2	0.5	0	0.5	0	0	0	0	0	650	432
5.100792972	2.714675185	7.246607479	0.592553408	0.296510359	0.88471571	0	0.845453759	0	0	20.01368632	1072.32
6	4	9	0.8	0.25	1	0	1	0	0	20	1068

5.907690655	4.076962064	3.938489561	0.52126565	0.267311113	0.808934741	0	0	0	0	600.0257336	609
5.396907876	6.203312634	0.509755451	0	0.495208258	0	0	0	0	0	599.9981434	610
6	4	7	0.8	0.25	1	0	1	0	0	700	524
6.016449677	3.99750964	9.00576557	0.803112502	0.280788808	1.011195749	0	0.998720824	0	0	649.9904446	658
6.502930725	4.192856486	5.568657745	2.774738329	0.408086965	1.786565536	1.980253183	3.597870846	0.101585956	0	19.99179318	1175
5.91	4.07	3.92	0.51	0.27	0.82	0	0	0	0	600	609
8.487195706	0	2.017547942	0	1.479180706	1.016964564	0	0	0.152110175	0	19.99903287	978
6	2.75	4	0.4	0.45	1	0	0	0	0	20	1020
4.540765372	2.574140005	2.138732376	1.402271711	0.410532063	2.032851528	0	1.08453485	0.137563988	0	20.00214094	1011.6
5.41	2.52	5.33	0.68	0.19	1.35	1.33	1.35	0.62	0	650	603
5.994463507	3.988021221	7.019647251	0.800352636	0.243002745	1.002139799	0	0.99887672	0	0	749.9977903	381
5.977888647	2.002356146	4.007708652	0.985214138	0.51143754	0	0	0	0	0	600.003385	615
4.578	2.898	2.867	1.479	0.517	2.072	0	1.144	0.388	0	750	528.3
4.682355656	2.894540018	2.855513913	1.461847308	0.468589692	2.066271748	0	1.064631814	0.272146365	0	20.00081874	905.5
6	4	11	0.8	0.25	1	0	1	0	0	650	692
5.795847121	4.206327819	3.922706929	0	0.401818663	0.702482206	1.495406391	0	0	0	19.99150156	871
5.999255666	3.006206721	10.00177701	0.786646556	0.303801979	1.006105857	0	1.005597904	0	0	600.0108078	580
6.502766908	4.208271832	5.550130019	2.784535341	0.417353432	1.807201692	1.996256673	3.581428424	0.079291075	0	750.0048247	749
6.015023571	4.000740948	7.016286155	0.786198985	0.232966176	0.999444523	0	1.003840654	0	0	19.99967305	1130
6.64	1.89	3.73	4.05	0.2	0	0	1.01	0	0	20	1085
6.006141667	4.007575077	8.994694989	0.794241818	0.247249483	0.976980788	0	0.984848089	0	0	20.01366874	1068

6	4	9	0.8	0.25	1	0	1	0	0	700	577
9.526723379	0	0	0.746078918	0	0	0	0	0	1.415364851	20.0029612	880
5.92	2.12	3.88	2.03	0.1	0	0	0	0	0	600	571
3.2	0	0	0.5	0	6.5	0.5	0	0	0.5	20	960
4.990767668	0	1.306483154	0.440241267	0	6.510536418	1.310506011	0	0	0.246323821	500.0049332	720
7.997596746	0	2.196251792	0	0.8071096	0	0	0	0	1.004442633	19.99639034	1028
5.4	6.2	0.5	0	0.5	0	0	0	0	0	600	610
5.38671814	6.201968612	6.407384666	0	0.501713683	0	3.998843517	1.596988963	0	0	649.9852148	842
5.993373762	4.005705987	10.99236741	0.781951179	0.233724576	1.000480849	0	1.002597225	0	0	749.9909568	451
3.198160167	0	0	0.500184339	0	6.503475817	0.494602403	0	0	0.492216953	20.00195845	960
6	4	7	0.8	0.25	1	0	1	0	0	750	381
5.996484865	2.000184184	4.016764373	1.003269274	0.497808995	0	0	0	0	0	550.0082941	635
4.543	2.567	2.134	1.403	0.419	2.048	0	1.089	0.129	0	20	1011.6
4.743	2.744	5.709	0.799	0.326	2.113	0	1.202	0.131	0	750	473.2
5.989189435	3.010531529	9.999604448	0.806815007	0.300283184	1.000297561	0	1.009382838	0	0	699.9948396	385
5.39323078	6.206116763	0.510309995	0	0.509312801	0	0	0	0	0	19.99160782	1003
8	0	2.2	0	1.4	0	0	0	0	1	20	1020
4.739786142	2.748129315	5.703362754	0.790777796	0.328436872	2.115449666	0	1.196930568	0.126289617	0	750.0023205	473.2
8.7	0	0	0.76	0	0	0	0	0	0.78	20	825
5.000260911	0	1.32517659	0.442742562	0	6.501867668	1.312446171	0	0	0.213884822	19.98593339	960
8.7	0	0	0.5	0	0	0	0	0	0.5	20	750

4.741698569	2.74496996	5.71495157	0.790817793	0.346923873	2.102939826	0	1.189858114	0.142581109	0	20.00791663	967.7
4.671	2.885	2.849	1.465	0.461	2.074	0	1.067	0.277	0	750	555.6
9.286518146	0	0	2.007432641	0	0	0	0	0	0	20.00170865	860
4.547	1.873	4.998	0.771	0.303	1.04	0	0.969	0	0	20	1038.45
5.997301251	2.990212363	9.995557067	1.003773005	0.257569886	0.990778347	0	1.008696059	0	0	700.0135564	492.5
6.010035329	0	0	0	0	0	0	0	0.01361636	3.993548802	20.00361396	925
6.000961208	2.995377247	9.995655038	0.796908279	0.302221338	0.995212514	0	1.012557561	0	0	19.99105393	992
5.439116149	2.389753579	5.386658858	0.662776768	0.26132314	0.843515135	0	0.81251152	0	0	20.00115675	1057.49
8.69	0	0	0.72	0	0	0	0	0	0.78	20	826
5.985519157	3.735925362	3.992815558	0.397865528	0.453109076	0.514753562	0	0	0	0	20.0085766	972
8.011593298	0	2.189189367	0	1.406159356	0	0	0	0	1.005931013	19.99690454	1020
6.509633761	4.204127809	5.558220602	2.78896793	0.417546119	1.772462638	1.981104856	3.581841897	0.089228983	0	700.0034115	874
4.588184913	2.734621035	5.788990597	0.764997831	0.436698021	0.672146864	0	1.085135173	0.145621307	0	700.0158602	643.1
8.494550809	0	2.003991361	0	1.499623653	0	0.211033019	0	0.151142276	1.001503018	19.99636388	952
7.8	0	2.2	0	1.4	0	0	0	0	1	500	847
6.64	1.89	3.73	4.05	0.2	0	0	1.01	0	0	550	813
5.418522782	6.199865028	6.389422891	0	0.508225449	0	3.987791564	1.602088636	0	0	599.9804033	1017
6.85	0	0	0	0	0	0	0	0	3.6	20	910
4.598	2.73	5.787	0.771	0.436	0.676	0	1.084	0.139	0	700	643.1
5	0	1.32	0.45	0	6.5	1.32	0	0	0.22	600	660
